# A Review of the Scale and Sustainability of the Consumption and Trade of Anuran Species in Africa

**DOI:** 10.1002/ece3.73148

**Published:** 2026-02-27

**Authors:** Sandra Owusu‐Gyamfi, Lauren Coad, Hannah N. K. Sackey, Zachary Braithwaite, Daniel Korley Attuquayefio, Yaa Ntiamoa‐Baidu

**Affiliations:** ^1^ Centre for Biodiversity and Conservation Research University of Ghana Accra Ghana; ^2^ Department of Animal Biology and Conservation Science University of Ghana Accra Ghana; ^3^ Center for International Forestry Research Bogor Indonesia; ^4^ University of Oxford Oxford UK

**Keywords:** amphibian trade, bushmeat, frog consumption, frog meat

## Abstract

Within Africa, collection and trade of anurans is often recorded as single‐site case studies, making it difficult to accurately understand the scale of use, its livelihood importance, and impact on species. We conducted a systematic review to: compile literature on anuran uses in Africa; identify the species and ecoregions involved; and identify gaps and opportunities for monitoring anuran utilisation. From an initial pool of 3335 articles, 85 studies on anuran use were reviewed. We augmented this with data from records on levels of anuran trade within CITES from 2012 to 2021, IUCN redlist, UNdata and WILDMEAT databases. We found 131 amphibian (124 anuran) species belonging to 18 families and 42 genera used within Africa. About 31.5% of species are used as food and 2.4% used in traditional medicine. Another 23.4% are used in multiple ways. Larger‐bodied species, including Hoplobatrachus occipitalis and Pyxicephalus edulis, are most preferred as food, whereas smaller and colourful ones (mostly in the Mantellidae family) are traded as pets. The use of anurans as food and traditional medicine is concentrated in Guineo‐Congolian and Guineo‐Sudanian ecoregions, whilst Madagascar and the Indian Ocean dominates the international pet trade. Wild populations of anuran species are collected mainly by local men and sold to intermediaries to supply food and pet markets. African countries import frog legs more than they export possibly, to supply locally based international restaurants. We identified the inability of most international databases to accurately capture the extent of anuran use with literature review identifying 28 additional species missed by these platforms. Also, there are few scientific studies that quantify the impacts of use on anurans in Africa. Synthesis and applications: We recommend that anuran species collection and trade be incorporated into national biodiversity monitoring plans.

## Abstract 2

This review article synthesises available data from literature, CITES, IUCN, UNdata and WILDMEAT to highlight the extent of Africa's involvement in anura utilisation. The review also makes suggestions to address unregulated harvesting of wild anuran species, especially as food in Africa.

## 1 Introduction

There are nearly 9000 species of amphibians described, of which 7898 are frogs and toads (AmphibiaWeb 2025; Ceríaco et al. 2014; Calderon and Stábeli 2011). Their wide distribution and the ability of some species to persist in human‐modified landscapes make them easy targets for collection and use (Neveu 2009).

It is likely that anurans have been consumed as food by humans since the ‘hunter–gatherer’ period of human history (see Whyte and Compton 2020). Kyselý (2008) reports archaeological findings from the Czech Republic which suggest that the European common frog ( *Rana temporaria* ) was used as food in prehistoric times (3000–2800 bce) and provided a convenient source of protein. There is also evidence of the consumption of frogs by many African communities (Das 2012; Mohneke 2011; Neveu 2009; Efenakpo et al. 2016). At the turn of the 20th century, international trade of live anurans had a new chapter when the African clawed frog ( *Xenopus laevis* ) began being used for human pregnancy testing (van Sittert and Measey 2016) in Europe and North America (Gurdon and Hopwood 2000). By the 1990s, demand surged for the colourful dendrobatid frogs (poison frogs) mostly driven by hobbyists from the Global North (Carpenter et al. 2014).

Recent estimates of the global trade in amphibians (Hughes et al. 2021) suggest that 17% (1215) of all amphibian species are traded, and of these, 80% are anurans. Most of the amphibian species were sold as pets (66%) or for research and breeding purposes (55%). However, over a third (33%; 415 species) were traded for food, and a further 10% for medicine or in pharmacological research. Although Hughes et al. (2021) provided a comprehensive review of the international and online trade, studies are still lacking on the levels of national and local use (but see Akinyemi and Efenakpo 2015; Angulo 2008).

Updated records and literature on global anuran use are needed to inform conservation policy. However, Africa's contribution to such studies is low, as data on the use and trade of any form of commodity are scarce on the continent (Buyonge and Kireeva 2008; Mintah et al. 2022). The absence of databases that contain trade levels, species involved, and the economic contributions is a major limitation to improving regulation and management of wildlife use and trade. Whilst this review consolidates existing literature and data on anuran use in Africa, it relies on the scanty information available on the subject in the continent. In that regard, this review produces a reference material which identifies the species involved and their threat status, hotspots for specific uses, and investigates the human dimensions and the motives for use, which can be a springboard for future research.

## 2 Methods

### 2.1 International and Regional Databases

We searched four databases for records of use and trade of African anurans. WILDMEAT database (https://www.wildmeat.org/), the IUCN Red List (2025), the CITES Trade Database (https://trade.cites.org/) and the UNdata Database (https://data.un.org/Default.aspx). Descriptions of the searches conducted are provided in Data S1. We then combined the results to compile a table of used species for each ecoregion of Africa (CEPF 2022; Droissart et al. 2018; Gorel 2019; RCMRD 2023), their Red List status, their main uses, and whether they are threatened by use (using the IUCN Red List Use and Threats classification). We further used data from the CITES Trade and the UNdata Databases to discuss key trends in anuran imports to and exports from Africa. We supplemented these data with a systematic literature review (see below), to add additional species and to include use data.

### 2.2 Systematic Literature Review

We conducted a systematic literature review, following the guidelines of Collaboration for Environmental Evidence (CEE 2013) which involved searching for articles in academic journals and web engines, screening results and extracting relevant data for synthesis. The snout‐vent length (SVL) of used species was obtained from AmphibiaWeb (2025).

The lead author (S.O.‐G.) searched three directories (Google Scholar, Google Web Engine and Scopus) to identify relevant academic and grey literature using appropriate search strings (Appendix 1 and Data S2) and English as the search language. There was no restriction of the reviewed data to a defined timeframe. Searches were conducted between 25 May and 15 December 2023.

The first 150 articles obtained from Google Web engine, Google Scholar and Scopus for each search string were considered for screening. We found many articles to be irrelevant after the first ~120 documents. Next, article titles and abstracts were reviewed against the eligibility criteria (Data S1). This was followed by the reading of the full content if the article passed the eligibility criteria. A consistency check of the eligibility screening process was completed using Cohen's Kappa analysis (conducted by S.O.‐G., H.N.K.S. and Z.B.). This resulted in a Kappa score of 0.64, suggesting sufficient agreement between reviewers (McHugh 2012).

## 3 Results

**FIGURE 1:**
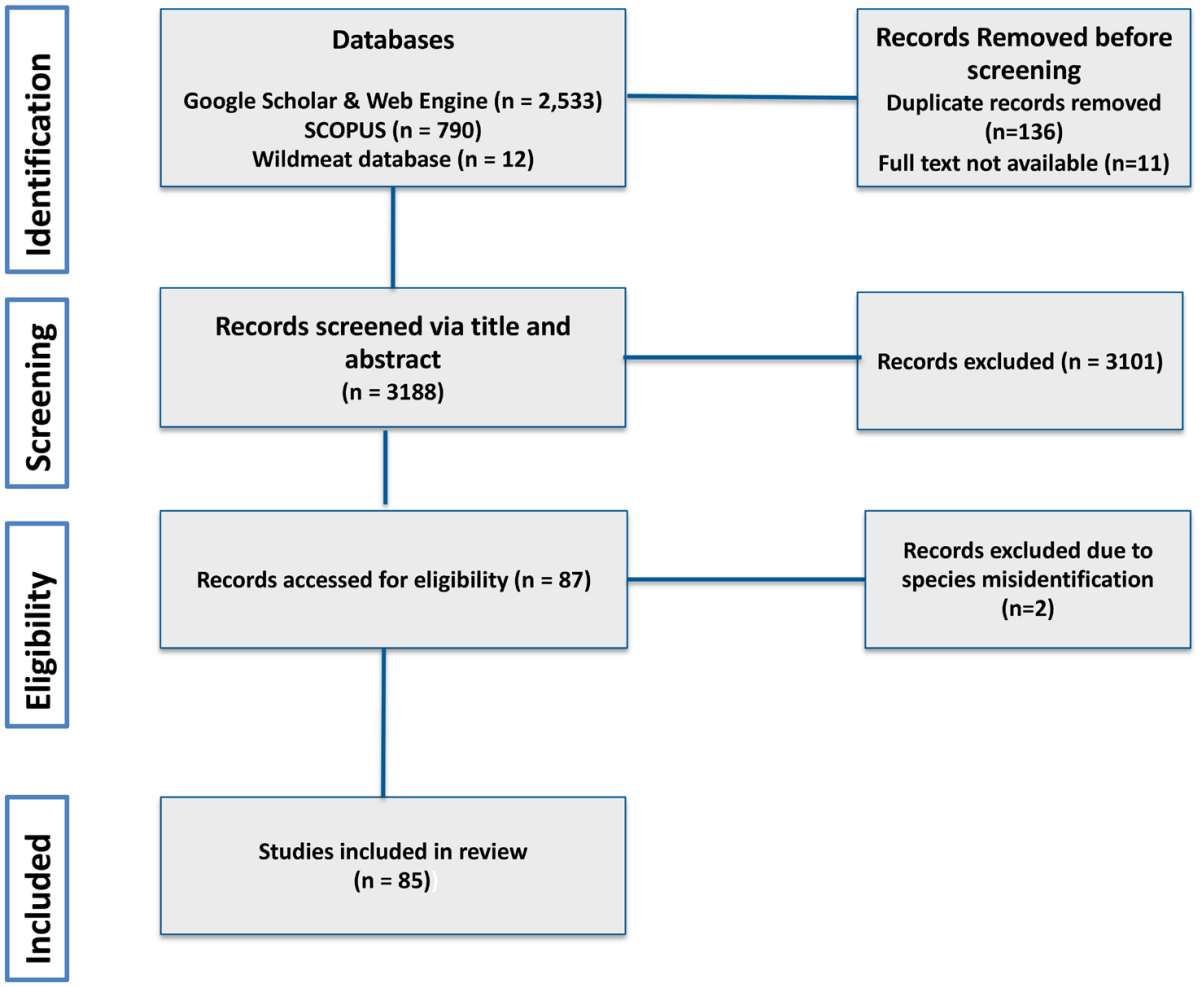
PRISMA flowchart for records assessed from literature.

We reviewed 3335 articles (Data S2). After applying our inclusion and exclusion criteria and removing repetitions, we narrowed them to 85 articles. We used these (Figure 1) to explore the characteristics, drivers and impacts of anuran use in Africa.

We found that 131 amphibian species from 42 genera and 18 families (Data S3) are used across Africa. Additionally, five records from five genera and five families were not identified to species level. Most of the used species are anurans (frogs = 113 and toads = 11); few are Caudata (*n* = 3) and Gymnophiona (*n* = 4). Thus, anurans formed the main basis of the review.

**FIGURE 2:**
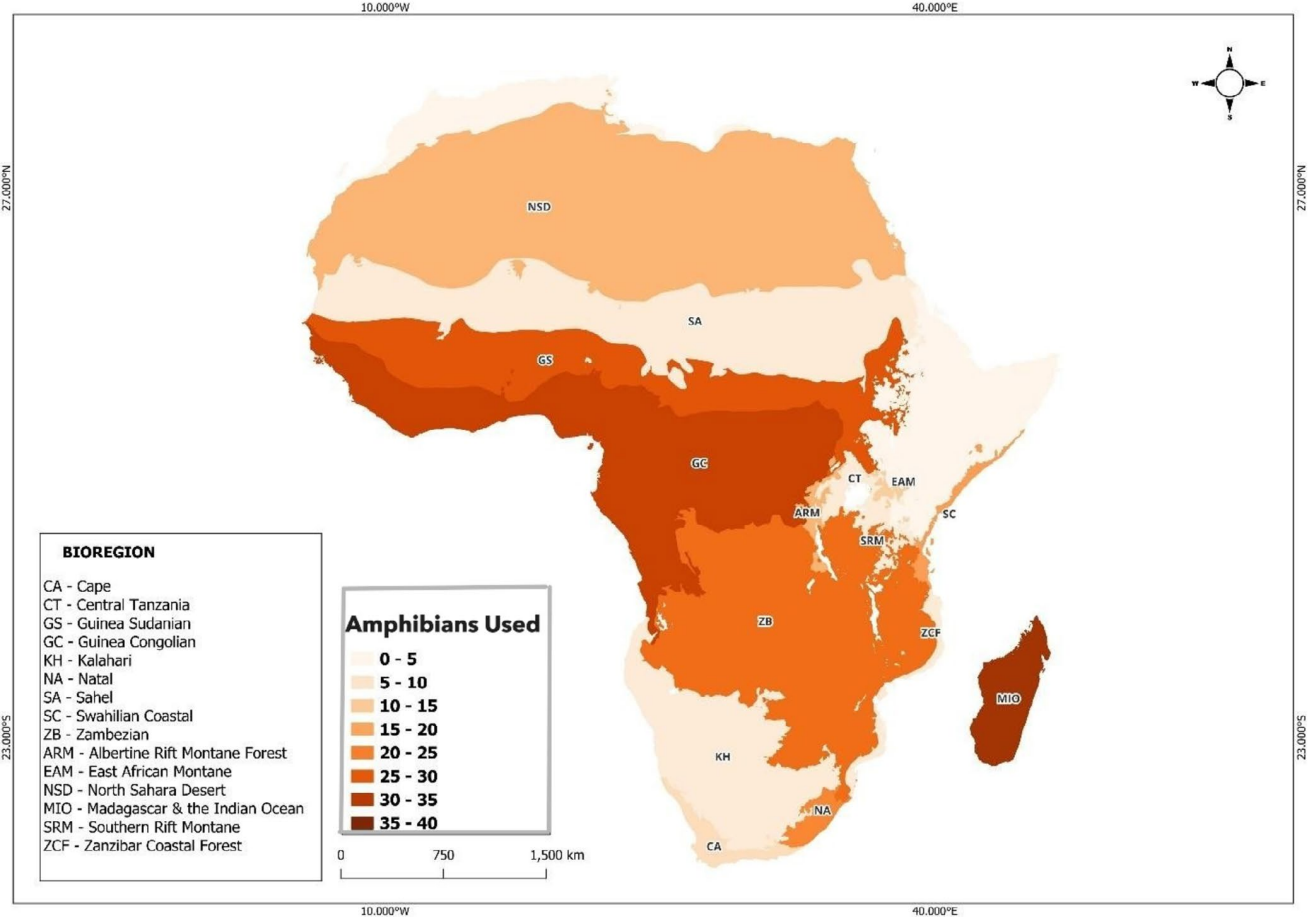
Number of amphibian species exploited in various biogeographic regions in Africa. The map shows all the 131 amphibian species used.

The African ecoregions with the most use are Madagascar and the Indian Ocean (*n* = 39), Guineo‐Congolian (*n* = 33) and Guineo‐Sudanian (*n* = 28; Figure 2). One of the used species, the Indian bullfrog ( *Hoplobatrachus tigerinus* ), is not native to Africa but has been introduced and is now consumed in Madagascar. Of the 124 anuran species recorded, the majority (*n* = 95) are Least Concern, with 11 species classified as Vulnerable, nine as Endangered, four as Near Threatened, two as Critically Endangered, and one as extinct in the wild. Only two species are Data Deficient (IUCN 2017a, 2017b, 2013). All species classified as Data Deficient, Vulnerable or above (*n* = 25) are restricted to three countries or less, and 56% of these are found only in Madagascar and the Indian Ocean.

The snout‐vent length (SVL) of used anurans ranges between 16 and 320 mm. For anuran species used as food (*n* = 28), their sizes range from 16 to 140 mm; for food and other purposes, including traditional medicine and pets (*n* = 23), their sizes range from 16 to 320 mm. Anurans used as pets (*n* = 50) range from 17 to 103 mm, but 80% are < 50 mm. For anurans used in traditional medicine (*n* = 3), they range from 60 to 116 mm.

Almost half (46) of the 96 used anuran species are listed as threatened by overexploitation (IUCN 2025). Twenty‐one species are threatened only by collection for pet trade, 18 only by consumption, six by pet trade and consumption, and one by use in traditional medicine. Every used conruid (slippery frogs) and half of dicroglossids (fork‐tongued frogs) are threatened by use. Two species— *Kassina decorata* and *Xenopus fischbergi* —did not have a purpose but are threatened by use (IUCN 2016a, 2016b). For the non‐anurans, two Gymnophiona ( *Geotrypetes seraphini* and *Herpele squalostoma* ) and one Caudata ( *Salamandra algira* ) are threatened by pet trade (IUCN 2021a, 2021b, 2017a, 2017b).

There is very little evidence to suggest a wide use of anurans as pets in Africa. The databases and literature reviewed suggest that demand for African anurans, specifically frogs, for the pet trade was from Asian, European and North American countries. Colourful and small‐sized frog species are the main target for international trade (see Data S3). The bright colours which protect species from predation in the wild (Vences et al. 2004) are prized characteristics for the pet trade.

Frogs for the pet trade come predominantly from Madagascar, which was the second highest global amphibian exporter between 2012 and 2021 (https://tradeview.cites.org/en/overview). The trade of frogs as pets is lucrative and has encouraged the establishment of private captive breeding facilities in Madagascar to help meet the high demand (Mattioli et al. 2006). Mattioli et al. (2006) calculated the price of a captive‐bred *Mantella aurantiaca* at $70 compared to the $20 to $55 offered for wild‐caught individuals. Online trading platforms, such as Willow Reptiles (2024) and Josh's Frogs (2024) now sell captive‐bred individuals of the species for between $85 and $150.

Collection of wild frog species in Madagascar for the pet trade involved multiple actors. Rabemananjara, Raminosoa, et al. (2008) reported that collectors were local farmers with knowledge of the species on demand, who could each collect between 100 and 300 individuals per day, depending on the season. These were then sold to intermediaries who resold to accredited exporters. This system was not rigid as intermediaries sometimes acted as collectors and exporters also.

Every frog species exported from Madagascar is endemic to the country. The status of 56% ranges from Critically Endangered to Near Threatened (Data S3). Granting private breeding facilities permission to export frogs should help reduce pressure on wild populations. Nevertheless, all live individuals exported (*n* = 70,589) reported in the CITES database (2012 to 2021) came from the wild. Compared with the 233,893 individuals of *Mantella* exported between 1994 and 2003 (Rabemananjara, Bora, et al. 2008), the current figure could possibly be a positive sign that trade regulations may be effective. However, it could also indicate serious declines in wild populations or a reduction in monitoring efforts.

The use of anuran species for food is more prevalent in West Africa and Cameroon (Guineo‐Congolian biome), than in other parts of the continent. However, this may reflect biases in research focus as much as true geographical differences in use. For example, we obtained 41 publications for Western Africa compared to 19 for the rest of Africa (some of which were also on pet trade). Mohneke et al. (2009) and Mohneke et al. (2011) were the first to document the use of anurans in West Africa. This might have encouraged others to build on these studies, as many of these later publications related to the same countries that the earlier authors studied, that is, Benin, Burkina Faso and Nigeria.

**FIGURE 3:**
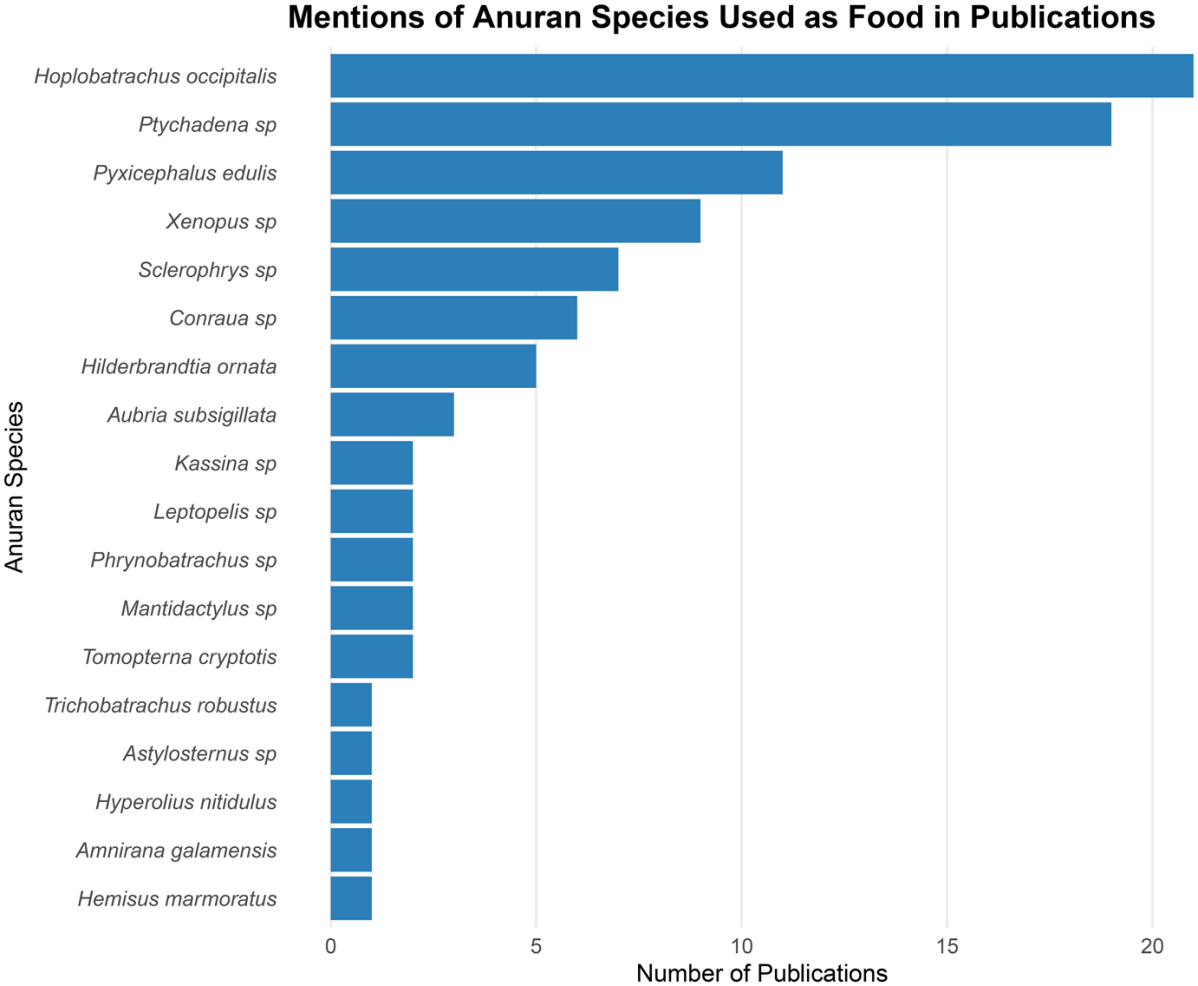
Anuran species and number of publication mentions.

Although some small‐sized frog species, such as *Kassina decorata* , are eaten by humans, the most commonly mentioned species in human diets in Africa are the ‘larger frogs’ especially *Hoplobatrachus occipitalis* and *Pyxicephalus edulis* (see Mohneke et al. 2009; Blé et al. 2016; Gansa et al. 2021; Sackey et al. 2022). At least 21 publications—the highest for a singular species—mention the use of *H. occipitalis* (Figure 3). These species live close to human settlements. The combination of these two factors increases their exposure to hunters. Smaller‐sized frogs are targeted to meet shortfalls in demand (Mohneke 2011). Local consumers eat the entire frog after extracting the guts (Salome Ibietela and Amuzie 2017).

The trade of frogs as food is highly localised in Africa. As a result, it is not often recorded in national or international statistics or databases. However, national trade volumes are likely to be significant (Mohneke 2011). For example, Akinyemi and Efenakpo et al. (2015) surveyed 100 consumers in two markets of Ibadan City, Nigeria. They estimated that 126,672 frogs were eaten by respondents annually.

A combination of factors influences the use of anurans in Africa. Increasing human populations and reduced access to wild mammals (due to either enforcement or declining populations) are creating the need to find alternatives to meet protein deficits (Akinyemi and Efenakpo 2015). Blé et al. (2016) found that a ban on the consumption of bushmeat in Côte d'Ivoire was the major catalyst for more people to use frogs to compensate for the lack of other wild meat options. Nutritional value (Efenakpo et al. 2016) and its ‘chicken’ or ‘fishy’ taste were major determinants of frog consumption in West Africa (Casimir et al. 2022). Omoniyi et al. (2012) also established that frog meat was a cheaper protein source, and this influenced the demand in the Ondo State in Nigeria. Similarly, Efenakpo et al. (2016) found that just under 35% of people in Ibadan, Nigeria (*n* = 100), ate frogs because it was cheap, while the rest consumed them for its nutritional value. Several studies have found that frog consumers were mainly from rural and peri‐urban areas and from low‐income groups in West Africa (see Onadeko et al. 2011; Akinyemi and Efenakpo 2015; Sackey et al. 2022). However, Blé et al. (2016) reported that in Côte d'Ivoire frog consumers could be both rural and city dwellers, and belonged to any social class, sex or age‐group. Tradition and custom may also drive use. Phaka et al. (2022) found that traditional healers and traders in South Africa set the quantity of each species to be collected depending on what charms and concoctions were to be made for clients. In some cases, religion may influence the decision to use anurans. For instance, Sanusi et al. (2016) reported that 93.3% of the study subjects (*n* = 90) in Nigeria's Sokoto State did not eat frogs as they were Muslims. According to Omoniyi et al. (2012), some Islamic groups considered frogs as unclean and not to be eaten. However, Efenakpo et al. (2016) did not record such religious restrictions in Ibadan—Nigeria, where Muslims ate and traded frog meat.

Frogs are more likely to be eaten than toads. Toads tend to be used for traditional medicine, because secretions from their parotoid or skin glands are believed to heal diseases and neutralise scorpion stings (Mohneke 2011; Efenakpo et al. 2016). There is scientific evidence to support the healing properties of toads. Studies have documented the anti‐inflammatory and cancer‐fighting properties, as well as the therapeutic potential of toad toxins (see Qi et al. 2018). The healing abilities of African toads remain to be validated, as most studies covered Southeast Asia and Australia. Notwithstanding, toads can still be found on people's plates, for example in Burkina Faso, where people remove the skin before drying to remove the toxins (Mohneke et al. 2009).

Most collectors of frog meat in Africa are rural dwellers who live close to collection sites (Mohneke et al. 2009; Onadeko et al. 2011; Rabemananjara, Raminosoa, et al. 2008). Mohneke et al. (2009 and 2011) have reported active frog collection by dedicated hunters (including women) in Benin, Chad and Nigeria to supply various markets in Nigeria. Such cross‐border trade has also been reported by Howard (2013) between Ghana and Burkina Faso, where the trade route is through the Feo and Soe communities in the Bongo District of northeastern Ghana. Many commercial collectors were uneducated (Gansa et al. 2021) and traded to supplement their income (Sackey et al. 2022). In some instances, frog hunting is even more profitable than fishing, to which Mohneke et al. (2009) attributed as a motivation for many fishermen to switch occupations in Benin. A two‐man team could catch between 200 and 1500 frogs per night in Mallanville‐Benin. These were then sent to Ibadan‐Nigeria by the collectors themselves or via intermediaries. At frog trading depots in Ibadan, a seller usually bought between six and 17.5 bags of frogs per week from harvesters (Akinyemi and Efenakpo 2015). Using an approximated count of 1000 individuals per bag (see Mohneke 2011), this translates to 6000 to 17,500 individuals per seller per week. This figure was reported not to be enough to meet the demand of traders, according to Akinyemi and Efenakpo et al. (2015). In Madagascar, frog collectors supplied continental restaurants directly. Jenkins et al. (2009) reported that 21 frog collectors supplied an average of 249 frogs per week to a single restaurant in Alaotra Mangoro Region in Madagascar.

The involvement of men and women in the trade varied. Mohneke et al. (2011), reported that women prepared and traded frogs in Burkina Faso. However, Efenakpo et al. (2015) observed only men trading in Ibadan, Nigeria.

Many species consumed in Africa, such as the large‐sized *Hoplobatrachus occipitalis* , are at risk of local extinction (Mohneke 2011). Cox et al. (2008) further indicate that range‐restricted species are also vulnerable to extinction. Thus, endemic species such as the vulnerable *Conraua robusta* which has an estimated area of occurrence of 17,303 km^2^ (IUCN 2019) and several traded *Mantella* species are threatened by use. The use of anuran species is an issue for ecosystem integrity. Studies from India and Bangladesh demonstrate that the hunting of species including the Jerdon's bullfrog ( *Hoplobatrachus crassus* ) and Indian skipper frog ( *Euphlyctis cyanophlyctis* ) has resulted in ecological release and an increase in agricultural pests and mosquitoes (Grano 2020). However, our literature review did not find any studies on the impact of hunting on frog populations and ecosystems in Africa.

## 4 Discussion

Our results demonstrate both the use of anuran species in Africa and the significant data gaps that exist in our understanding of their importance as a source of food and income, and the ecological impacts of their use.

While international trade databases provide some data on anuran trade, most data available are concentrated in international pet trade and focus on colourful, small‐bodied species, predominantly from Madagascar. Almost all species used for food are eaten and sold locally, and this phenomenon is likely not captured by international trade databases. Another challenge in using the IUCN database is that it does not always provide site‐specific information to help narrow the information down to areas where the use is occurring. This limits the ability to direct conservation efforts to the right geographic area. Databases that quantify wild meat trade using site‐level data on hunting, consumption and market sales, such as WILDMEAT (Willis et al. 2022), have the potential to capture this use. However, our searches of the WILDMEAT database (which currently includes data for Central Africa) resulted in only three relevant records of frog use, which we know from our literature review does not reflect the reality in many areas. Furthermore, although this review did not include information on trade as reported by importing countries on the CITES trade database, upon a cursory review, we found instances where the exporting countries delayed in reporting. As at the time of the review, no exporting country had updated their trade records with CITES for 2021, while importing countries reported 1203 individual frogs received from Madagascar. Again in 2019, USA received 1143 individuals of *Mantella betsileo* , which Madagascar was yet to report. There are also instances in which the importer's reported number of trades exceeded that of the exporter's (e.g., see rows 272, 286, 318, 333, of CITES trade data 2012–2021). Such discrepancies and lack of reporting could lead to underestimation of the actual trade volume, leading to insufficient monitoring of populations.

The lack of anurans in surveys may be because studies of wild meat harvests have traditionally focused on mammals (see Groom et al. 2023). Additionally, frogs may also be caught more opportunistically by women and children (see Mohneke et al. 2011) and therefore are not recorded by most traditional hunting surveys. Frog consumption may also be localised. This seems to be the case, for example in Ghana, from the lead author's personal experience and data from Sackey et al. (2022), frog consumption is prevalent within the northern regions. As the WILDMEAT project also provides tools and methods for wild meat data collection, these methods should highlight the need to collect data on a range of taxa in addition to mammals, as well as capturing opportunistic harvests by women and children.

The IUCN Red list Use classification is an important source of information on species use. However, it often depends on dedicated expert groups and primary data on the use of anurans for food that is biased towards a few regions and countries, such as Benin and Burkina Faso. Nevertheless, our literature review identified an additional 28 species which were not yet included in the Red List as being used. The Red List also highlights the potential ecological impacts of anuran use, with 47.9% of used species currently threatened by it. However, we found few scientific studies that quantified the impacts of use. Thus, this should be a priority for future research.

Information on frog legs trade from the UNdata database provides some unexpected results, showing that overall, Africa imports more frog legs than it exports. This may be driven by trends in economic migration. An estimated one to two million Chinese migrants currently live in Africa (Siu and McGovern 2017; Cissé 2021). China is a major consumer of frogs (Grano 2020). It would be relevant to explore further the final destinations of these frog leg imports.

A unifying challenge for all data sources was species identification. UNdata does not provide a species ID, and more generally, frog leg misidentification is high. For example, 98% of randomly tested 209 frog specimens from French supermarkets were misidentified (Ohler and Nicolas 2017). Similar challenges are faced by surveys of markets in West Africa, where the processes of frying and smoking (Salome Ibietela and Amuzie 2017) destroy species' morphological features. For example, Mohneke et al. (2011), relied on photo guides to identify traded species. Advances in hand‐held DNA testing units may provide a more accurate way of identifying species sold at markets as costs decrease (Doi et al. 2020).

Considering that most trade databases are likely not capturing a high percentage of anuran usage due to their international trade focus, monitoring of anuran consumption and trade should be incorporated into national biodiversity monitoring plans. These plans can then inform strategies to mitigate impacts on anuran populations, including the pros and cons of captive breeding. Randomised pre‐screening of frog legs imports for infectious diseases such as chytridiomycosis (or Bd) needs to be introduced to prevent the introduction of deadly amphibian pathogens. West African anuran species have until now been spared from the deadly Bd (Penner et al. 2013). However, the fact that this fungus has been found among anuran species in Madagascar (Kolby et al. 2014, 2015) should alert other African countries to take necessary precautions to prevent their spread.

Although Africa's dependence on wildlife as a major source of protein and income is well‐documented, the lack of inclusion of less charismatic groups such as anurans has limited our understanding of the full scale of wildlife use. This review confirmed the ‘silent’ collection and use of anurans in Africa at local and national levels, potentially at a scale comparable to or greater than that of the documented international trade. This review should act both as an alarm and a call to action to prevent the depletion of anurans at regional and continental levels.

## Author Contributions

**Sandra Owusu‐Gyamfi:** conceptualization (equal), data curation (lead), formal analysis (lead), funding acquisition (supporting), investigation (lead), methodology (lead), project administration (lead), visualization (supporting), writing – original draft (lead). **Lauren Coad:** conceptualization (equal), funding acquisition (lead), methodology (equal), resources (lead), supervision (equal), validation (equal), writing – review and editing (equal). **Hannah N. K. Sackey:** formal analysis (equal), validation (equal), writing – review and editing (equal). **Zachary Braithwaite:** data curation (lead), formal analysis (equal), investigation (lead), methodology (lead), visualization (lead), writing – original draft (lead). **Daniel Korley Attuquayefio:** supervision (equal), validation (equal), writing – review and editing (equal). **Yaa Ntiamoa‐Baidu:** conceptualization (lead), funding acquisition (lead), supervision (lead), writing – original draft (equal), writing – review and editing (equal).

## Funding

This work was supported by the United States Agency for International Development, UK Research and Innovation's Global Challenges Research Fund, Trade, Development and the Environment Hub Project (ES/S008160/1), Carnegie Corporation of New York, Rufford Foundation and Synchronicity Earth.

## Conflicts of Interest

The authors declare no conflicts of interest.

## Supporting information


**Data S1:** Eligibility criteria of documents for review and detailed extraction processes from each database.

**Data S2:** Results of search strings obtained from each directory.

**Data S3:** Used species list.

**Data S4:** Anuran species used and the reporting sources.

## Data Availability

All data used in this review are found in the Supporting Information.

## Appendix 1 Search Terms Used to Search Online Directories

**Table:**

Term to describe Anurans		Term to describe use
Frog OR Frogs OR Anura OR Amphibian	AND	(trade OR export OR commercial trade)
		(“traditional medicine” OR “medicine”)
		(“Unsustainable use” OR “harvesting” OR “exploitation”)
		(meat OR wildmeat OR bushmeat OR “wild meat” OR “frog meat” OR frogmeat OR frog bushmeat OR “frog bush meat”)
		(pet OR pet trade OR international trade OR “pet” OR “pet trade”)
(“frogmeat*” OR “frog meat*” OR “frog consumption*” OR “frog use”)		
