# Supplementary material for: A Review of the Scale and Sustainability of the Consumption and Trade of Anuran Species in Africa
Source: Ecol Evol. 2026 Feb 27;16(3):e73148. doi: 10.1002/ece3.73148 (PMC12949087; doi:10.1002/ece3.73148)
Supplement: Supplementary file 1 — Data S1: Eligibility criteria of documents for review and detailed extraction processes from each database. [file ECE3-16-e73148-s004.docx]

**Data S1**

1. **WILDMEAT**

The WILDMEAT Use Database holds data on wild meat consumption, hunting offtakes and market sales within one database and in one standardised format. In WILDMEAT database, we were specific with our search term, entering just “amphibians” in the site’s search engine.

1. **Data Extraction Process from IUCN Red List**

The IUCN assesses how close species are to becoming extinct and records their assessment in the ‘Red List of Threatened Species’ (IUCN, 2025). After assessment, species are given one of nine Red List Categories which indicate the level of extinction risk they face e.g., Least Concern, Near Threatened, etc. The Red List provides further data including information on the use and/or trade of assessed species.  Table below details the data extraction process. We downloaded the data from version 2024-2 of the IUCN Red List.

**Table S1 Data extraction process from the IUCN Red List website. Search filters allow you to filter species by various characteristics and download option lets you choose which information about species you download.**

| **Search Filters** | |
| --- | --- |
| **Filter** | **Option** |
| Taxonomy | Amphibia |
| Land Regions | ‘North Africa’ and ‘Sub-Saharan Africa’ |
| All other filters were unchanged. | |
| **Download Options** | |
| **Option** | **Reason** |
| Assessments | Provides a csv file detailing the Red List Category and a summary of information on each species |
| Countries | A file listing the countries each species is or may be found in. |
| Taxonomy | A file listing the taxonomic ranks each species belongs to. |
| Threats | A file listing the factors that threaten each species. |
| Usetrade | A file listing the ways each species is used. |

If individuals of a species or part of them are harvested for use, the Red List assigns the species use and trade categories according to how it is used, such as ‘Food – human’ or ‘pets/display animals, horticulture’ (IUCN, 2020). The scale of use was also recorded - ‘International’, ‘National’, ‘Subsistence’ or a combination of them. A use was “Local livelihood- subsistence” if “harvesters made use of the species or its parts or products themselves” and “National” or “National commercial value” was when “parts or products of the species were sold/traded on a national basis i.e., within the country but away from harvested areas” (IUCN, 2020). If this scale data was not recorded for a species, we assigned them a scale of “Not given”. We defined species as used if they had a use and trade category. We also read the description associated with their use categories (use description) and decided to remove and edit some species based on this, Table S2.

**Table S2, species removed from or edited in the used species list because of their use description in the IUCN red list**

| **Species** | **Use description as stated in IUCN** | **Reason for removal or alteration** |
| --- | --- | --- |
| *Barbarophryne brongersmai* | This species is not found in the pet trade, but it has been present in a number of private collections since it was described and it breeds well in captivity (D. Donaire-Barroso pers. comm. 2020). | Removed. It is not found in the pet trade. |
| *Bufotes boulengeri* | This species has been observed for sale in at least one pet store in Madrid, but it is unknown how widely it is traded (I. Martinez-Solano pers. comm. 2020). | Removed. It is found in several North African countries, Spain and Italy. As it is found in Spain, it is most likely the traded species was collected there, not Africa. |
| *Duttaphrynus melanostictus* | This species is consumed by people in much of its range and it can often be found in the international pet trade. At current levels neither of these uses appear to be a threat to the population. Secretions from the auricular and skin glands are also used in traditional Chinese medicine (Bick et al. 2002, Chow et al. 2003, Dasgupta 2003). It is not utilized for food or found in the pet trade in India; however, the skin of this species is used for musical instruments in some tribal areas of Odisha, although this is not considered to be a major threat (India Red List Assessment Workshop August 2020). | Removed. It has been introduced to Madagascar, but the description does not mention any use there. |
| *Xenopus longipes* | There are no records of this species being utilized. Experts were concerned about the increasing international interest in this species and worried the species would be harvested for international trade in the near future, if it is not already occurring. However, this is based on one incident of an animal dealer contacting a Cameroonian field assistant for collection, which turned out to be merely prospective (T. Doherty-Bone pers. comm. February 2017). | Removed. There are no records of this species being utilized. |
| *Boulengerula taitana* | In the 2004 assessment it was recorded to be in the international pet trade in very small numbers, but there have been no further records since. | Removed. No further records of trade for 21 years. |
| *Hoplobatrachus tigerinus* | “... It is considered a pest in Madagascar, where it was originally introduced as a source of human food, and it is still harvested for this purpose, but not at a level to contain the spread of the species. …” | Edited-  1) In the Red List it has the following use categories: “Food - Human” and “Research”. we removed “Research”. Its Red List use description says it is used for food and research, however it does not mention collection for research in Madagascar, only Asia.  2) Scale of use of “Food - Human” changed from “International”, “National” and “Subsistence” to only “Subsistence”. Its use description does not explicitly say it is used for food at “International” or “National” level in Madagascar. |
| *Pleurodeles waltl* | This species is frequently used in laboratory studies of embryogenesis (Busack and Donaire 2014). It is sometimes offered for sale in the pet trade (D. Escoriza pers. comm. 2020). D. Donaire-Barroso (pers. comm. 2020) reports that it is "widely traded" in the UK and US, where it is popular due to its hardiness, and is also traded in France, the Netherlands, Belgium, Germany, Russia, Spain and Japan. This species was traditionally used as a pest control agent in wells, but this is no longer the case following the widespread importation of North American mosquitofish (Gambusia spp.) for this purpose. | Edited- in the Red List, it has the following use categories: “pets, display animals/horticulture”; “Research” and “other”.  we removed “other”. “other” refers to use as a pest control agent in wells. We removed this because the use description says it is no longer used this way. |

If a species was used unsustainably (i.e., the use was, is, or may be a driver of decline for the species), it was recorded in the Red List threats section (IUCN, 2022). If a wild amphibian species was threatened due to use, it was given the threat category “5.1 Hunting and Trapping terrestrial animals”. The threat category “5.1.1” indicates the hunting or trapping of the terrestrial animal species is intentional i.e. the species is the target. We defined  a species as threatened by use if it had a use category, had the threat category “5.1.1” and threat 5.1.1 was described as “Ongoing”. We identified the use categories that threaten these species (threatening uses), if a threatened species only had one use category, that was the threatening use. If a threatened species had more than one use category, we read its threat description, we define threatening uses as use categories described as a threat, or words to that effect, including those described as “not a major threat”. We did not assign the Gymnophiona species *Crotaphatrema lamottei* as used because it does not have a use category, although it is subject to threat category 5.1.1 because locals kill this species, possibly because of its snake-like appearance.

One of the authors (Z.B.) cleaned the data manually or using R code (v 4.3.1; R Core Team 2023). To create the figures representing the Red List data, he wrote R code (v 4.3.1; R Core Team 2023) and modified the R code used for figures in Coad et al. (2021) (v 4.3.1; R Core Team 2023).

We looked at the Red List ‘Countries’ data, to see if any used species had been introduced from outside Africa.  *Hoplobatrachus tigerinus* has threat category 5.1.1 but is only in Africa because of human introduction, so we do not recognise *H. tigerinus* as threatened in Africa.

We (Z.B.) found the total number of amphibian species in Africa on the IUCN Red List by choosing the following search filters: ‘Amphibia’ in the ‘Taxonomy’ filter; ‘North Africa’ and ‘Sub-Saharan Africa’ in the ‘Land Regions’ filter and leaving all other filters as their default selections (IUCN, 2025).

We (Z. B.) used Red List guidelines (IUCN, 2020) to assign use categories to species identified in the literature review that did not already have a use category.

1. **CITES**

CITES trade database is a free online resource managed by UNEP World Conservation Monitoring Centre which documents the trade in both fauna and flora species as reported by parties to the Convention on International Trade in Endangered Species (Robinson & Sinovas, 2018).

We downloaded Anuran trade records from the CITES Trade Database, choosing the most recent 10-year period of 2012 to 2021. We filtered the search terms selecting the order “Anura” in “Search by taxon”; and selecting all member states of the African Union (<https://au.int/en/member_states/countryprofiles2>) within the category of “exporting countries” and maintaining the default for the options: “importing countries,” “source,” “purpose,” and “trade terms.” A second search was conducted for anuran imports into Africa, repeating the same as earlier but selecting member states in the “importing countries” category and leaving the “exporting countries” with the default options.  We used the exporting countries’ reported trade figures for all calculations. Where the “Unit” information was blank, it was assumed the traded species were individuals as CITES recommends (see https://trade.cites.org/cites_trade_guidelines/en-CITES_Trade_Database_Guide.pdf).

1. **UNdata**

UNdata is the United Nations (UN) statistical system which provides statistical data on various themes including agriculture, environment, and trade (UNdata, 2024). Within the directory’s search engine, we entered the only allowable commodity trade term of “frog legs” and narrowed the countries by selecting only member states of the African Union. We did not limit the search to a particular timeframe.

1. **Inclusion And Exclusion Criteria of Obtained Documents**

**Our inclusion criteria required that the document should be/have:**

- frogs or anuran used (as food, medicine, pet, or research) as the main focus;
- an article, book, report or thesis;
- written in English;
- full and accessible text online; and
- focused or inclusive of Africa or an African country.

**We eliminated the document if the publication:**

- was an online newspaper or blog;
- was in a language other than English;
- could be accessed only through its abstract and or had to be purchased if it was not within the University of Oxford’s online library catalogue;
- had no bearing with the main topic under review; and
- provided geographic coverage that does not include Africa.

**e. Biogeographic Zones**

“North Sahara” in the map represents all areas above the Guinea savanna. “Zanzibar inhambane coastal forest mosaic" was simply named "Zanzibar coastal forest"

**References**

- IUCN. 2025. The IUCN Red List of Threatened Species. Version 2024-2. [https://www.iucnredlist.org](https://www.iucnredlist.org/).
