# Supplementary material for: A Review of the Scale and Sustainability of the Consumption and Trade of Anuran Species in Africa
Source: Ecol Evol. 2026 Feb 27;16(3):e73148. doi: 10.1002/ece3.73148 (PMC12949087; doi:10.1002/ece3.73148)
Supplement: Supplementary file 3 — Data S3: Used species list. [file ECE3-16-e73148-s003.docx]

Amphibian species used in Africa as reported in literature, IUCN and CITES with the corresponding SVL of adults (obtained from AmphibiaWeb - https://amphibiaweb.org/; Baraj et al. 2010 & 2011; Blackburn et al. 2020; Channing & Rödel, 2019; Evans et al. 2015; Erismis, 2011; Kowalski et al. 2020; Kalayci et al. 2019; Amor et al. 2010; Rödel & Bangoura, 2004; Ernst et al. 2015; Tinsley et al. 1979). *Data source = Literature Review (L), IUCN (I), CITES (C). LC = Least concern, DD = Data deficient, EW = Extinct in the wild, EN = Endangered, CR = critically endangered, NT = near threatened, VU = vulnerable. ** Data Source = Literature Review (L), IUCN (I). The articles which mention the species can be found in Supplementary S8.

| **Common name** | **Scientific name** | **Biogeographical region(s)** | **Adult SVL (mm)** | **Data source*** | **Type of use**** | **IUCN Red List** |
| --- | --- | --- | --- | --- | --- | --- |
| **Arthroleptidae** |  |  |  |  |  |  |
| Hairy frog | *Trichobatrachus robustus* | Guineo-Congolian | 90-130 | L, I | Food | LC |
| Night spirit frog | *Leptopelis spiritusnoctis* | Guineo-Congolian | 30-49 | L | Pet (international trade) | LC |
| Savanna forest tree frog | *Leptopelis bufonides* | Guineo-Congolian | 29-41 | L | Food & Traditional medicine | LC |
| Night frogs | *Astylosternus* sp*.* | Guineo-Congolian | - | L | Food |  |
| Bagamoyo Forest Treefrog | *Leptopelis argenteus* | Swahilian coastal | 29-33 | I | Pet (international trade) | LC |
| Bocage's tree frog | *Leptopelis bocagii* | Zambezian & East African montane | Up to 58 | I | Pet (international trade) | LC |
| Savanna tree frog | *Leptopelis viridis* | Guineo-Congolian | 33-48 | L | Food | LC |
| Yellow-spotted tree frog | *Leptopelis flavomaculatus* | Zambezian | 44-70 | L | Pet (international trade) | LC |
| Common forest tree frog | *Leptopelis notatus* | Guineo-Congolian | 42-74 | I | Food & Traditional medicine | LC |
| Ruby-eyed tree frog | *Leptopelis uluguruensis* | Zambezian | 28-48 | L | Pet (international trade) | NT |
| Vermiculated Tree Frog | *Leptopelis vermiculatus* | Zambezian | 39-85 | I | Pet (international trade) | EN |
| Mountain Egg Frog | *Leptodactylodon bicolor* | Guineo-Congolian | - | I | Food | NT |
| **Bufonidae** |  |  |  |  |  |  |
| Penton's toad | *Sclerophrys pentoni* | Guineo-Sudanian | 54-95 | L | Food & Traditional medicine | LC |
| African common toad | *Sclerophrys regularis* | Guineo-Sudanian | 62-130 | L, I | Food, Pet trade & Traditional medicine | LC |
| Flat-backed toad | *Sclerophrys maculata* | Guineo-Sudanian | 38-60 | L, I | Food & Traditional medicine | LC |
| Sub-Saharan toad | *Sclerophrys xeros* | Guineo-Sudanian | 52-87 | L | Food & Traditional medicine | LC |
| African giant toad | *Sclerophrys superciliaris* | Guineo-Congolian | 116-128 | L, I, C | Food, Pet trade & Traditional medicine | LC |
| Togo Toad | *Sclerophrys togoensis* | Guineo-Congolian | 62-64 (only males) | L, I | Traditional medicine | LC |
| Common toad | *Bufo bufo* | Sahel | 52-116 | L | Traditional medicine | LC |
| African red toad | *Schismaderma carens* | Kalahari & Natal | 88-92 | L | Traditional medicine | LC |
| Kihansi spray toad | *Nectophrynoides asperginis* | Zambezian | 16-22 | I, C | Ex-situ production | EW |
| **Conrauidae** |  |  |  |  |  |  |
| Abo slippery frog | *Conraua crassipes* | Guineo-Congolian | Up to 81 | L | Food | LC |
| Goliath frog | *Conraua goliath* | Guineo-Congolian | 150-320 | L, I, C | Food & pet trade | EN |
| Togo slippery frog | *Conraua derooi* | Guineo-Congolian | Similar to other *Conraua* sp | I | Food & Pet trade | CR |
| Cameroon slippery frog | *Conraua robusta* | Guineo-Congolian | 120-140 | L, I | Food | VU |
| **Dicroglossidae** |  |  |  |  |  |  |
| African crowned bullfrog | *Hoplobatrachus occipitalis* | Guineo-Sudanian | 52-160 | L, I | Food & Fishing bait (Food - animal) | LC |
| Indian bullfrog | *Hoplobatrachus tigerinus* | Madagascar & the Indian Ocean | 120-170 | L, I, C | Food & Research | LC |
| **Hemisotidae** |  |  |  |  |  |  |
| Marbled snout-burrower | *Hemisus marmoratus* | Guineo-Sudanian | 22-49 | L, I | Food & Pet trade | LC |
| **Hyperoliidae** |  |  |  |  |  |  |
| The decorated running frog | *Kassina decorata* | Guineo-Congolian | 21-35 | L | Food | VU |
| Brown running frog | *Kassina fusca* | Guineo-Sudanian | 29-33 | L | Food & Traditional medicine | LC |
| Senegal running frog | *Kassina senegalensis* | Guineo-Sudanian | 25-49 | L, I | Pet (international trade) | LC |
| Madagascar Reed frog | *Heterixalus madagascariensis* | Madagascar & the Indian Ocean | 35-40 | L, I | Pet (international trade) | LC |
| Plain reed frog | *Hyperolius nitidulus* | Guineo-Sudanian | 23-32 | L | Food | LC |
| Lime reed frog | *Hyperolius fusciventris* | Guineo-Congolian | 18-28 | L | Pet (international trade) | LC |
| Tinker reed frog | *Hyperolius tuberilinguis* | East African montane & Natal | 25-35 | L | Pet (international trade) | LC |
| Spotted reed frog | *Hyperolius puncticulatus* | Zanzibar Coastal Forest | 21-37 | L, I | Pet (international trade) | EN |
| Riggenbach's reed frog | *Hyperolius riggenbachi* | Guineo-Congolian | 27-40 | L | Pet (international trade) | LC |
| Argus Reed Frog | *Hyperolius argus* | Zambezian | 27-34 | I | Pet (international trade) | LC |
| Parker's reed frog | *Hyperolius parkeri* | Swahilian coastal | 21-24 | I | Pet (international trade) | LC |
| Reed frog | *Hyperolius pictus* | Zambezian | 23-29 | I | Pet (international trade) | LC |
| Variable reed frog | *Hyperolius concolor* | Guineo-Sudanian | 24-40 | I | Pet (international trade) | LC |
| Spotted Reed Frog | *Hyperolius substriatus* | Swahilian coastal | - | I | Pet (international trade) | LC |
| Red-legged Kassina | *Hylambates maculatus* | Swahilian coastal | 55-65 | L, I | Pet (international trade) | LC |
| Common reed frog | *Hyperolius viridiflavus* | East African montane | - | I | Pet (international trade) | LC |
| Whitebelly Reed Frog | *Heterixalus alboguttatus* | Madagascar & the Indian Ocean | 30-33 | I | Pet (international trade) | LC |
| Painted Reed Frog | *Hyperolius marmoratus* | Zambezian & Swahilian coastal | Up to 33 | I | Pet (international trade) | LC |
| Betsileo Reed Frog | *Heterixalus betsileo* | Madagascar & the Indian Ocean | 18-29 | I | Pet (international trade) | LC |
| Boettger's Reed Frog | *Heterixalus boettgeri* | Madagascar & the Indian Ocean | 22-29 | I | Pet (international trade) | LC |
| Rutenberg's Reed Frog | *Heterixalus rutenbergi* | Madagascar & the Indian Ocean | 25-27 | I | Pet (international trade) | LC |
| Fornasini's Spiny Reed Frog | *Afrixalus fornasini* | Swahilian coastal | 30-40 | I | Pet (international trade) | LC |
| Kivu Banana Frog | *Afrixalus orophilus* | Albertine rift montane | 19-27 | I | Pet (international trade) | LC |
| **Mantellidae** |  |  |  |  |  |  |
| Golden mantella | *Mantella aurantiaca* | Madagascar & the Indian Ocean | 19-31 | L, I, C | Pet trade & Research | EN |
| Malagasy poison frogs | *Mantella* sp. | Madagascar & the Indian Ocean | - | L, C | Pet (international trade) |  |
| Madagascar poison frog | *Mantella baroni* | Madagascar & the Indian Ocean | 22-30 | L, I, C | Pet (international trade) | LC |
| Bernhard's mantella | *Mantella bernhardi* | Madagascar & the Indian Ocean | 19-22 | L, I, C | Pet (international trade) | VU |
| The brown mantella | *Mantella betsileo* | Madagascar & the Indian Ocean | 18-26 | L, I, C | Pet (international trade) | LC |
| Cowan's mantella | *Mantella cowanii* | Madagascar & the Indian Ocean | 22-29 | L, I, C | Pet (international trade) | EN |
| Eastern golden frog | *Mantella crocea* | Madagascar & the Indian Ocean | 17-24 | L, I, C | Pet (international trade) | VU |
| Madagascan brown frog | *Mantella ebenaui* | Madagascar & the Indian Ocean | - | I, C | Pet (international trade) | LC |
| Blue-legged mantella | *Mantella expectata* | Madagascar & the Indian Ocean | 20-26 | L, I, C | Pet (international trade) | EN |
| Haraldmeier's mantella | *Mantella haraldmeieri* | Madagascar & the Indian Ocean | 21-27 | L, I, C | Pet (international trade) | EN |
| Folohy golden frog | *Mantella laevigata* | Madagascar & the Indian Ocean | 22-29 | L, I, C | Pet (international trade) | LC |
| Madagascan mantella | *Mantella madagascariensis* | Madagascar & the Indian Ocean | 20-27 | L, I, C | Pet (international trade) | VU |
| Marojejy mantella | *Mantella manery* | Madagascar & the Indian Ocean | 23-29 | I, C | Pet (international trade) | VU |
| Black-eared mantella | *Mantella milotympanum* | Madagascar & the Indian Ocean | 19-30 | L, I, C | Pet (international trade) | CR |
| Guibé's mantella | *Mantella nigricans* | Madagascar & the Indian Ocean | 27-28 | L, I, C | Pet (international trade) | LC |
| Parker's golden frog | *Mantella pulchra* | Madagascar & the Indian Ocean | 21-25 | L, I, C | Pet (international trade) | NT |
| Green golden frog | *Mantella viridis* | Madagascar & the Indian Ocean | 22-30 | L, I, C | Pet (international trade) | EN |
| Madagascar bright-eyed frog | *Boophis madagascariensis* | Madagascar & the Indian Ocean | 60-103 | L, I, C | Pet (international trade) | LC |
| White-lipped Bright-eyed Frog | *Boophis albilabris* | Madagascar & the Indian Ocean | 43-81 | I | Pet (international trade) | LC |
| Goudot's Bright-eyed Frog | *Boophis goudotii* | Madagascar & the Indian Ocean | 50-87 | I | Food & Pet trade | LC |
| Imerina Bright-eyed Frog | *Boophis microtympanum* | Madagascar & the Indian Ocean | 27-42 | I | Pet (international trade) | LC |
| Angel's Madagascar Frog | *Boehmantis microtympanum* | Madagascar & the Indian Ocean | 60-80 | I | Food | VU |
| Gray Madagascar frog | *Mantidactylus guttulatus* | Madagascar & the Indian Ocean | 100-120 | L, I | Food | LC |
| Grandidier's Madagascar frog | *Mantidactylus grandidieri* | Madagascar & the Indian Ocean | 75-108 | L, I | Food | LC |
| **Microhylidae** |  |  |  |  |  |  |
| Sambava tomato frog | *Dyscophus guineti* | Madagascar & the Indian Ocean | 60-95 | L, I, C | Pet (international trade) | LC |
| Madagascar tomato frog | *Dyscophus antongilii* | Madagascar & the Indian Ocean | 60-105 | L, I | Pet trade & establishing ex-situ production | LC |
| Antsouhy tomato frog | *Dyscophus insularis* | Madagascar & the Indian Ocean | 40-50 | L, I, C | Pet (international trade) | LC |
| Tomato frogs | *Dyscophus* sp. | Madagascar & the Indian Ocean | - | L | Pet (international trade) |  |
| Marbled rain frog | *Scaphiophryne marmorata* | Madagascar & the Indian Ocean | 32-44 | L, I, C | Pet (international trade) | VU |
| Boribory rain frog | *Scaphiophryne boribory* | Madagascar & the Indian Ocean | 49-59 | L, I | Pet (international trade) | VU |
| Madagascar rain frog | *Scaphiophryne madagascariensis* | Madagascar & the Indian Ocean | 41-56 | L, I | Pet (international trade) | NT |
| Malagasy rainbow frog | *Scaphiophryne gottlebei* | Madagascar & the Indian Ocean | 20-40 | L, I, C | Pet (international trade) | EN |
| Green burrowing frog | *Scaphiophryne spinosa* | Madagascar & the Indian Ocean | 40-48 | L, I, C | Pet (international trade) | LC |
| Banded Rubber Frog | *Phrynomantis bifasciatus* | Zambezian & Natal | Up to 75 | I | Pet trade & Establishing ex-situ production | LC |
| **Phrynobatrachidae** |  |  |  |  |  |  |
| Warty river frog | *Phrynobatrachus francisci* | Guineo-Sudanian | 16.1-24.5 | L | Food | LC |
| Natal dwarf puddle frog | *Phrynobatrachus natalensis* | Guineo-Sudanian | 25-31 | L | Food | LC |
| Itombwe River Frog | *Phrynobatrachus asper* | Guineo-Congolian | - | I | Food | VU |
| **Pipidae** |  |  |  |  |  |  |
| Andre's clawed frog | *Xenopus andrei* | Guineo-Congolian | 22-36 | I | Food | LC |
| *Fraser's Clawed Frog* | *Xenopus fraseri* | Guineo-Congolian | 16-19 | I | Food & pet trade | DD |
| Müller's clawed toad | *Xenopus muelleri* | Guineo-Sudanian | 38-60 | L, I | Food | LC |
| Volcano clawed frog | *Xenopus amieti* | Guineo-Congolian | 35-49 | L, I | Food | VU |
| African clawed frog | *Xenopus laevis* | Guineo-Congolian, Cape & Natal | 45.6-147 | L, I | Food & research | LC |
| Fischberg's clawed frog | *Xenopus fischbergi* | Guineo-Sudanian | 51.7-62.6 | L | Food | LC |
| Tropical clawed frog | *Xenopus tropicalis* | Guineo-Sudanian | 28-55 | L, I | Food | LC |
| Peters' platanna | *Xenopus petersii* | Zambezian | - | I | Food | LC |
| Bouchia Clawed Frog | *Xenopus pygmaeus* | Guineo-Congolian | - | I | Food | LC |
| Uganda clawed frog | *Xenopus ruwenzoriensis* | Guineo-Congolian | 36-39 | I | Food | DD |
| Kivu clawed frog | *Xenopus vestitus* | Albertine rift montane | - | I | Food | LC |
| Mwanza Frog | *Xenopus victorianus* | Guineo-Congolian, Guineo-Sudanian & Albertine rift montane | 35 | I | Food & research | LC |
| De Witte's clawed frog | *Xenopus wittei* | Guineo-Congolian & Albertine rift montane | 45-61 | I | Food | LC |
| Merlin's dwarf gray frog | *Pseudhymenochirus merlini* | Guineo-Congolian | - | I | Food | LC |
| Western dwarf clawed frog | *Hymenochirus curtipes* | Guineo-Congolian, Cape & Natal | 24-33 | L, I | Pet (international trade) | LC |
| Congo dwarf clawed frog | *Hymenochirus boettgeri* | Guineo-Congolian | Up to 35 | I | Pet trade & Establishing ex-situ production | LC |
| **Ptychadenidae** |  |  |  |  |  |  |
| Broad-banded grassland frog | *Ptychadena bibroni* | Guineo-Sudanian | 34-55 | L | Food | LC |
| South African sharp-nosed frog | *Ptychadena oxyrhynchus* | Guineo-Sudanian | 40-64 | L | Food | LC |
| Spotted-throated ridged frog | *Ptychadena pumilio* | Guineo-Sudanian | 25-36 | L | Food | LC |
| Victoria Grassland Frog | *Ptychadena aequiplicata* | Guineo-Congolian | - | I | Food | LC |
| Sierra Leone Grassland Frog | *Ptychadena superciliaris* | Guineo-Congolian | - | I | Food | LC |
| Dakar grassland frog | *Ptychadena trinodis* | Guineo-Sudanian | 42-60 | L | Food | LC |
| Mascarene ridged frog | *Ptychadena mascareniensis* | Guineo-Sudanian | 43-68 | L, I | Food, Pet trade & Traditional medicine | LC |
| Grass frogs | *Ptychadena* sp. | Guineo-Congolian | - | L | Food |  |
| Schilluk ridged frog | *Ptychadena schillukorum* | Guineo-Sudanian | 43-49 | L | Food | LC |
| Central grassland frog | *Ptychadena tellinii* | Guineo-Sudanian | 30-47 | L | Food | LC |
| Tournier's rocket frog | *Ptychadena tournieri* | Guineo-Sudanian | 33-43 | L | Food | LC |
| African ornate frog | *Hildebrandtia ornata* | Guineo-Sudanian | 38-67 | L, I | Food & Pet trade | LC |
| **Pyxicephalidae** |  |  |  |  |  |  |
| Maluti River Frog | *Amietia vertebralis* | Natal | Up to 120 | I | Food & Traditional medicine | LC |
| Edible bullfrog | *Pyxicephalus edulis* | Guineo-Sudanian | 83-120 | L, I | Food, Pet trade & Other household goods | LC |
| African bullfrog | *Pyxicephalus adspersus* | Zambezian & Natal | 151-254 | L, I | Food & pet trade | LC |
| _ | *Aubria* sp. | Guineo-Congolian | - | L | Food |  |
| The brown ball frog | *Aubria subsigillata* | Guineo-Congolian | 65-95 | L | Food | LC |
| Parry's Bullfrog | *Pyxicephalus angusticeps* | Zambezian | - | I | Food & pet trade | LC |
| Common sand toad | *Tomopterna cryptotis* | Guineo-Sudanian | 38-64 | L | Food & Traditional medicine | LC |
| Red Sand Frog | *Tomopterna luganga* | Central Tanzania & Southern rift montane | 36-53 | I | Pet (international trade) | LC |
| **Ranidae** |  |  |  |  |  |  |
| Yellow-striped frog | *Amnirana galamensis* | Guineo-Sudanian | 62-77.4 | L, I | Food | LC |
| North African green frog | *Pelophylax saharicus* | North Saharan Desert | 39.7-104.5 | L, I | Pet trade & Research | LC |
| Bedriaga's Frog | *Pelophylax bedriagae* | North Saharan Desert | 42-96 | I | Food | LC |
| **Racophoridae** |  |  |  |  |  |  |
| Grey foam-nest tree frog | *Chiromantis xerampelina* | Zambezian | 43-90 | I | Pet (international trade) | LC |
| Peters' foam-nest treefrog | *Chiromantis petersii* | Zambezian | - | I | Pet (international trade) | LC |
| **Herpelidae** |  |  |  |  |  |  |
| Congo Caecilian | *Herpele squalostoma* | Guineo-Congolian | - | I | Pet (international trade) | LC |
| **Dermophiidae** |  |  |  |  |  |  |
| Gaboon Caecilian | *Geotrypetes seraphini* | Guineo-Congolian & Guineo-Sudanian | Up to 400 | I | Pet (international trade) | LC |
| São Tomé Caecilian | *Schistometopum thomense* | Guineo-Congolian | 129-350 | I | Pet (international trade) | LC |
| **Scolecomorphidae** |  |  |  |  |  |  |
| Lake Tanganyika Caecilian | *Scolecomorphus kirkii* | Central Tanzania & Southern rift montane | 163-485 | I | Pet (international trade) | LC |
| **Salamandridae** |  |  |  |  |  |  |
| The Algerian ribbed newt | *Pleurodeles nebulosus* | North Saharan Desert | 46-82.5 | I | Pet (international trade) | LC |
| Sharp-ribbed Salamander | *Pleurodeles waltl* | North Saharan Desert | Up to 31 | I | Pet (national and international trade) | LC |
| North African Fire Salamander | *Salamandra algira* | North Saharan Desert | Up to 200 | I | Pet (international trade) | VU |
